# Beyond Screen Time: A Scoping Review of Screen Exposure and Early Childhood Development

**DOI:** 10.3390/healthcare14193206

**Published:** 2026-09-28

**Authors:** Mshari Alghadier, Ragab K. Elnaggar, Abdulaziz Ghandurah, Nouf Alhisony

**Affiliations:** 1 Department of Health and Rehabilitation Sciences, College of Applied Medical Sciences, Prince Sattam Bin Abdulaziz University, Alkharj 11942, Saudi Arabia; r.elnaggar@psau.edu.sa (R.K.E.); z.ghandurah1@gmail.com (A.G.); noufalhisony@gmail.com (N.A.); 2 Rehabilitation Department, Ministry of the National Guard-Health Affairs, Riyadh 11426, Saudi Arabia

**Keywords:** screen exposure, screen time, early childhood development, language development, executive function, caregiver mediation, Saudi Arabia, scoping review

## Abstract

Background: Screen exposure in early childhood has evolved beyond traditional duration-focused “screen time” to encompass a multidimensional construct including content quality, context of use, co-viewing, caregiver mediation, and developmental trajectories. Understanding these dimensions is essential for comprehensive assessment, particularly in rapidly digitizing cultural contexts such as Saudi Arabia and the broader Middle East. Objectives: This scoping review aimed to systematically map and synthesize peer-reviewed evidence on multidimensional screen exposure and developmental outcomes in children aged 0–5 years, with specific focus on language, cognitive, and socio-emotional outcomes. Methods: This scoping review followed PRISMA-ScR guidelines. Systematic searches were performed in PubMed, Scopus, Web of Science, and Google Scholar (2016–2026). Inclusion criteria were primary empirical studies (randomized trials and observational designs), children aged 0–5 years (up to 72 months, provided primary exposure was assessed within the 0–5-year window), quantitative screen exposure measurement, and validated developmental outcome assessment. Data were extracted and synthesized narratively across the developmental domains. Results: Twenty-seven studies demonstrated that content quality, context of use, and caregiver behavior (including co-viewing and active guidance of screen use) consistently modified the strength of associations between screen exposure and early developmental outcomes. Language development was the most extensively studied domain; co-viewing emerged as a key protective factor, and longitudinal trajectory analyses identified heterogeneous exposure patterns with differing developmental implications across cognitive, socio-emotional, and physical domains. Conclusions: Screen exposure in early childhood is a multidimensional phenomenon requiring assessment tools that capture content, context, co-use, and caregiver mediation alongside duration. Findings highlight the need for culturally adapted measurement instruments in the Saudi Arabian context to support evidence-based early childhood practice and align with Vision 2030 objectives.

## 1. Introduction

Infancy and early childhood, defined as the period from birth to five years of age, represents a dynamic window of accelerated neurological, linguistic, cognitive, motor, and socio-emotional development. During this period, the brain exhibits heightened plasticity and sensitivity to environmental inputs, making it a critical timeframe for the establishment of foundational developmental trajectories [1,2,3]. Both biological maturation and experiential factors including caregiving quality, social interaction, physical activity, and access to stimulating learning environments have been documented to exert significant and lasting influences on child development outcomes [4,5].

Traditional approaches to children’s digital media use relied primarily on total daily screen time (ST); however, duration alone provides an incomplete account of developmental associations because screen exposure (SE) is multidimensional, encompassing content quality and type, context, caregiver mediation, device characteristics, age of onset, constancy, and developmental trajectories over time [6,7,8,9,10]. Constancy refers to the persistent or ambient presence of digital media in children’s daily lives, reflecting how screens are embedded within family routines, social interactions, and learning environments. This ecological perspective is reflected in the Dynamic, Relational, Ecological Approach to Media Effects Research (DREAMER) framework and related family media ecology models [11,12,13,14]. Content quality is also important, as child-directed educational programming may show different developmental associations from adult-directed entertainment or fast-paced, age-inappropriate content, while language variety, vocabulary complexity, and cultural relevance may further influence language-related outcomes [8,13,15,16].

The context in which SE occurs further shapes developmental associations. Joint media engagement, involving caregiver discussion and interaction, may create opportunities for learning, whereas background television can disrupt attention, reduce parent–child interaction, and displace play and other enriching activities; timing, such as screen use during meals or before bedtime, and solitary versus social use may also be relevant [17,18,19]. Caregiver mediation is another important dimension and includes active discussion, questioning, connections to real-world experiences, and restrictive rules concerning duration, content, and timing. Co-viewing without interactive scaffolding may provide limited benefit, while caregiver mental health, parenting stress, and broader family media habits are interconnected with children’s SE patterns [20,21].

Research on SE and early childhood development spans multiple domains, particularly language, cognition, and executive function. Meta-analytic evidence suggests that greater SE, especially passive or unsupported use is associated with smaller receptive and expressive vocabularies, delayed language milestones, and reduced parent–child verbal interaction. However, effects are generally modest and heterogeneous, highlighting the importance of exposure quality, content, and caregiver support rather than duration alone [15,22,23]. Longitudinal studies have linked greater ST or earlier exposure with less favorable attention and some executive function outcomes, including working memory and inhibitory control, in toddlers and preschool-aged children, while trajectory studies suggest that sustained high exposure across infancy and early childhood is associated with poorer later developmental outcomes than low or increasing exposure patterns [24,25].

Socio-emotional development, including emotional regulation, prosocial behavior, attachment security, and peer relationships, is also increasingly recognized as a screen-sensitive domain. Meta-analytic evidence reported small-to-moderate associations between SE and behavioral difficulties, including internalizing and externalizing problems [26]. Physical development is similarly relevant, as sedentary screen-based behaviors may displace active play that supports motor development and physical activity. In addition, pre-sleep device use and access to devices in the bedroom are associated with sleep disruption, which may partly contribute to observed relationships between screen exposure and developmental outcomes [10,27,28].

Saudi Arabia provides a salient context for studying SE in early childhood, as high smartphone access and social media use have made digital media increasingly embedded in family life from infancy [29,30] and household media context and quantity are associated with language outcomes in Saudi toddlers [31]. Yet, existing evidence on SE and early childhood development derives predominantly from Western contexts; research from Saudi Arabia and the MENA region remains sparse, despite the region’s distinctive media landscape and the prominence of early childhood development under Vision 2030.

This scoping review was conducted to systematically map and synthesize peer-reviewed evidence on the multidimensional nature of SE and its associations with early childhood developmental outcomes (0–5 years). By adopting a broad, inclusive approach consistent with scoping review methodology, the review aimed to identify the range of SE dimensions assessed in the literature, characterize developmental domains examined, summarize key findings across studies, and identify evidence gaps relevant to Saudi Arabia and the MENA region. The specific objectives were: (1) to describe the methodological and sample characteristics of included studies; (2) to map the SE dimensions and assessment approaches used; (3) to synthesize findings by developmental domain; and (4) to identify implications for the development of a multidimensional SE measurement tool for use with Saudi children in clinical and research settings. To organize the evidence, this review adopts a four-layer conceptual framework integrating SE dimensions, contextual moderators, proposed mediating mechanisms, and developmental outcomes (Figure 1).

## 2. Methods

### 2.1. Study Design and Reporting

This scoping review was conducted following the methodological framework proposed by Arksey and O’Malley [32]. The reporting of this review adheres to the Preferred Reporting Items for Systematic Reviews and Meta-Analyses extension for Scoping Reviews (PRISMA-ScR) checklist [33], and operationalized by the Joanna Briggs Institute (JBI) Manual for Evidence Synthesis [34] to ensure methodological rigor and transparency. The protocol for this review was registered retrospectively on the Open Science Framework (OSF) on (https://doi.org/10.17605/OSF.IO/UQKVD). No formal critical-appraisal tool was applied, and no quantitative results were pooled. The objective is to map the available evidence, characterize the literature, and identify gaps to inform future systematic reviews and primary research, not to evaluate or synthesize effect estimates.

### 2.2. Population, Concept, and Context

The eligibility framework was defined using Population, Concept, and Context (PCC). Studies were retained if the primary exposure assessment occurred within the 0–5-year window; studies whose follow-up assessments extended marginally beyond 60 months (e.g., to 72 months) were retained provided that the core SE measurement was conducted during the eligible early childhood period and the study was primarily focused on that age range. Studies focusing exclusively on clinical populations (e.g., children with diagnosed developmental disorders) were excluded unless they also reported community-based or normative-sample data. Screen exposure as a multidimensional construct encompassing duration of use, content quality (educational vs. entertainment), context of use (solo vs. co-viewing), interactivity (passive vs. active/interactive), and caregiver mediation (active mediation, co-viewing, restrictive mediation) was defined as the main concept. Outcomes of interest included developmental outcomes across three domains: language and communication development, cognitive development and executive function, and socio-emotional development and behavior. The geographic context was global, with MENA region tagged during extraction. No geographic restrictions were applied to the broader search, enabling comprehensive mapping of international evidence alongside regional data.

### 2.3. Search Strategy

PubMed, Scopus, Web of Science, and Google Scholar were searched for English-language publications dated January 2016 through July 2026. The date range was selected to capture contemporary smartphone and tablet exposure and cover the last ten years. Google Scholar was treated as a supplementary discovery source because its ranking and export functions are less reproducible than those of bibliographic databases. Search concepts were organized into three core blocks, with a fourth regional block used for targeted MENA searching. The full Boolean search strings for each database are available as Supplementary Materials.

### 2.4. Eligibility Criteria

Eligible studies examined any involvement of screen (ST and SE), including duration, content, context, interactivity, or caregiver mediation, in relation to developmental outcomes. Studies involving children older than 5 years, exclusively clinical or special populations without normative data, or SE linked only to non-developmental health outcomes such as obesity or vision problems were excluded. No geographic restrictions were applied, although particular attention was given to evidence from MENA region. Eligible study designs included randomized controlled trials and observational studies (cross-sectional, longitudinal, and experimental). Studies were required to include quantitative measurement of SE; qualitative and purely mixed-methods designs that did not report quantitative SE data were excluded. Reviews, opinion articles, editorials, conference abstracts without full text, non-English publications, studies published before January 2016, dissertations, theses, government reports, and other grey literature were excluded.

### 2.5. Study Selection

Records identified through database searches were imported into a reference management system and de-duplicated. Two independent reviewers (AG and NA) screened all titles and abstracts against the eligibility criteria. Studies were retained if either reviewer identified potential relevance. Disagreements were resolved through discussion, and a third reviewer (MA) was consulted when consensus could not be reached. Full-text screening was conducted for all studies passing title/abstract screening. Both reviewers independently assessed full-text eligibility, and reasons for exclusion were documented. Inter-rater agreement was assessed across all screened records (*n* = 964), with reviewers reaching consensus on 95% of title and abstract decisions. Discrepancies were resolved through discussion between the two reviewers, with no unresolved disagreements requiring arbitration by a third party.

### 2.6. Data Extraction and Synthesis

A standardized data extraction form was developed and piloted. Extracted data included: first author, publication year, country, study design, sample size, age range, SE measure, developmental outcome measure(s), and key findings. Data extraction was conducted by AG and NA, with cross-checking by RE. Discrepancies in extraction were resolved through team discussion. No contact with study authors was undertaken for missing data, consistent with the scoping review approach. Given the heterogeneity in study designs, measures, and populations, a narrative synthesis approach was adopted. Studies were organized by developmental domain (language and communication, cognitive development and executive function, socio-emotional development and behavior), and findings were synthesized thematically within each domain. Regional patterns, particularly from MENA, were highlighted and compared to findings from other geographic contexts.

Throughout this review, the term caregiver mediation refers exclusively to parenting behaviors related to screen use, including active co-viewing, content discussion, and restrictive rule-setting. This term is conceptually distinct from statistical mediation, which denotes a formally tested causal pathway. Where the included studies examined caregiver mediation in relation to SE outcomes, it functioned as a moderating variable, that is, a factor that modified the strength or direction of the association between SE and developmental outcomes, rather than a statistically established mediator. Moreover, the term ‘excessive SE’ throughout this review reflects thresholds defined by the original study authors, most commonly exceeding the AAP/WHO guideline of two hours per day for children aged 2–5 years; no independent threshold was applied by the reviewers.

## 3. Results

### 3.1. The Study Selection

The systematic search identified 1450 records. After removing 486 duplicates, 964 records underwent title and abstract screening; 767 were retained and sought for retrieval, of which 203 could not be obtained (see limitation section) resulting in 564 full-text articles assessed for eligibility. Twenty-seven primary empirical studies met all inclusion criteria and were included in the final synthesis. The study selection process is presented in the PRISMA flow diagram (Figure 2).

### 3.2. Characteristics of Included Studies

The 27 included studies examined children from birth through early childhood (0–5 years), with two studies retaining participants whose follow-up assessments extended marginally to 72 months, provided that primary SE measurement occurred within the 0–5 years window. The majority of studies focused on toddlers and preschoolers aged 1–5 years. Studies spanned diverse geographic contexts (Saudi Arabia, Australia, Canada, China, the United States, and others), and employed varied methodologies including longitudinal cohort studies, cross-sectional surveys, and experimental designs. Sample sizes ranged from small studies (*n* < 100) to large population-based cohorts (*n* > 1000). Developmental outcomes were assessed across three primary domains: (1) language and communication, including vocabulary and expressive and receptive language; (2) cognitive development and executive function, including attention, inhibitory control, and working memory; and (3) socio-emotional and behavioral development, including internalizing and externalizing problems, emotional regulation, and prosocial behavior. Of the 27 included studies, 17 (63%) examined language and communication outcomes, six (22%) examined cognitive development and executive function, and four (15%) examined socio-emotional and behavioral outcomes; each study was categorized according to its primary developmental domain. Measurement of SE varied substantially across studies, from parental questionnaires and time-use diaries to ecological momentary assessment and validated multidimensional instruments. Table 1 summarizes the included studies.

The two Saudi Arabian studies made important contributions. A study examined 288 preschool children (aged 1.5–5 years) in Saudi Arabia, assessing associations between technology use (smartphones, tablets, gaming devices) and socio-emotional and behavioral outcomes using the Arabic Children’s Behavior Questionnaire [35]. Another study investigated the home literacy and media environment of 85 Saudi toddlers (aged 12–36 months), examining associations between screen media quantity, content (target audience, genre, language variety), context (co-viewing, joint media engagement, background TV), and language development using standardized vocabulary and utterance measures [36]. 

### 3.3. Screen Exposure Dimensions

The most examined dimension was duration/quantity, assessed in all 27 studies, reflecting the historical emphasis on ST as a primary metric. However, 70% of studies also examined content quality or type, and two-thirds investigated contextual factors such as co-viewing and background exposure, consistent with the field’s evolution toward multidimensional frameworks [6,7]. More recent studies (published 2020–2024) were markedly more likely to examine multiple dimensions simultaneously.

Caregiver mediation (as a parenting practice; see Section 2), examined in 56% of studies, emerged as a notable modifier of association between SE and developmental outcomes. Studies distinguished between active mediation (discussing content, asking questions, connecting to real-world experiences) and restrictive mediation (setting time limits, content restrictions), with active mediation showing plausible positive effects, particularly for language development [19,20]. Device type, examined in 52% of studies, revealed differential associations by platform. Traditional television, particularly background TV showed more negative associations with language and attention compared to interactive touchscreen use, though the portability and constant accessibility of smartphones and tablets raised distinct concerns about displacement of parent-child interaction [11,37]. Longitudinal studies examining trajectories (30% of studies) identified heterogeneous patterns. Children following early-increasing trajectories demonstrated poorer cognitive and executive function outcomes compared to children with stable low exposure [38,39]. Table 2 summarizes the SE dimensions coded across the 27 included studies.

**Table 1:** Characteristics of the included studies, characterized by its primary developmental domain.

Authors (Year)	Country	Design	*n*	Age Range	Screen Exposure Measure	Developmental Outcome Measure	Key Finding
**Domain A: Language and Communication (*n* = 17)**							
Rayce et al. (2024) [40]	Denmark	Cross-sectional population survey	31,125	2–3 years	Parent report of normal-weekday mobile use: none, <1 h, 1–2 h or >2 h/day	Parent-reported Five to Fifteen Toddlers language-comprehension (7 items, α = 0.83) and expressive-language (5 items, α = 0.86) scales.	≥1 h/day mobile use was associated with poorer language scores and higher odds of comprehension and expressive-language difficulty. Frequent reading partly buffered comprehension risk but not expressive-language risk.
Kulkarni & Waknis (2024) [41]	India	Exploratory cross-sectional	60	1–4 years	Semi-structured parent questionnaire	Checklist for Assessment of Pragmatics of Preschoolers; communication functions, responses, conversation/interaction and contextual variation.	In the recommendation-compliant subgroup, greater ST was positively correlated with several pragmatic domains and total pragmatic score. Authors interpret supervised, limited screen use as potentially beneficial, but age/confounding and subgroup selection require caution.
Brushe et al. (2024) [18]	Australia	Prospective longitudinal cohort	220	12–36 months	LENA 16-h audio recordings; screen/electronic-noise segments manually coded in 5-min blocks and linked with parent activity diaries; sleep periods excluded.	LENA adult-word count, child vocalizations and conversational turns.	More ST was associated with fewer adult words, child vocalizations and conversational turns, with the largest effects at 36 months.
Gath et al. (2026) [42]	New Zealand	Prospective longitudinal cohort	6281	2–8 years	Parent reports	Short PPVT receptive vocabulary; parent oral-language/literacy communication; writing/numeracy tasks; DIBELS letter naming; SDQ peer problems/prosocial behavior.	Higher early SE was associated with lower later vocabulary, communication, writing, numeracy and letter fluency and more peer problems. Associations were reduced but remained after social-background adjustment.
Gath et al. (2023) [43]	New Zealand	Cross-sectional	84	3–5 years	Parent-reported daily use	CELF-P2 Recalling Sentences; digital story recall comprehension; Child-Parent Relationship Scale (CPRS) closeness/conflict; parent-rated quality interactions and shared reading.	More TV/electronic use was associated with lower language production, comprehension, shared reading, interaction quality and parent-child closeness. Reduced shared reading mediated screen-time associations with language; interaction quality mediated TV-closeness association.
Alroqi et al. (2023) [36]	Saudi Arabia	Cross-sectional/predictive observational study	85	1–3 years	Parent survey plus seven event-based diaries	JACDI-WG (12–16 months: receptive/expressive vocabulary) and JACDI-WS (17–36 months: words produced and mean length of three longest utterances).	In 12–16-month-olds, interactive joint media engagement and verbal co-engagement related to larger receptive vocabulary. In 17–36-month-olds, lower SE and more reading related to more expressive words; screen context and reading predicted mean length of utterance. Content effects were largely non-significant.
Pebriana et al. (2025) [44]	Indonesia	Cross-sectional descriptive/observational analytic	60	2–5 years	Questionnaire categorized screen-time intensity as high, moderate or low	KPSP developmental pre-screening questionnaire; speech-delay classification	No statistically significant association between screen-time intensity and speech delay was reported.
Stamati et al. (2022) [45]	Argentina	Cross-sectional parent-report study	253	2–48 months	Ad hoc parent questionnaire.	MacArthur-Bates Communicative Development Inventory Form II: lexical density and sentence use; parent-reported motor and language milestones (sitting, walking, object manipulation, words and sentences).	More screen-use hours were positively associated with reported lexical density and sentence use, while later initiation was associated with earlier milestone acquisition in several analyses. No general association with milestones was observed for total duration; content type did not differentiate outcomes.
Kanwal et al. (2023) [46]	Pakistan	Cross-sectional	318	2.5–5 years	Parent/caregiver questionnaire	ASQ-III communication/language domain and age-specific milestone items.	The paper reports poorer language milestone attainment among children with longer SE, especially the >9 h group, but provides limited inferential reporting.
Slobodin et al. (2024) [47]	Israel	Prospective longitudinal community study	179	0–3 years	Parent 24-h activity diary scored 0–5; screen score summed across devices	Official well-baby clinic developmental milestones in language/communication, motor, personal-social and fine/gross motor domains; referral records to child-development centers at 36 months	SE at 6 and 12 months was associated with language/communication deficits at 36 months in moderate- and high-SES families, but not low-SES families. SE at 24 months correlated with later motor problems; other moderated models were non-significant.
Takahashi et al. (2023) [48]	Japan	Prospective cohort	7097	1–4 years	Parent questionnaire asking typical daily ST	Japanese Ages & Stages Questionnaires, third edition (ASQ-III), five domains	Dose-response associations were strongest and most consistent for communication and problem-solving at both ages. Fine motor and personal-social associations appeared at age 2 but not age 4; gross motor was not associated.
Tiwari et al. (2025) [49]	India	Observational cross-sectional	120	6 moths–5 years	Caregiver structured questionnaire	Denver Developmental Screening Test II (DDST-II), assessed at enrollment and four-month follow-up.	The paper reports more language and social delays in the >2 h/day group and inverse associations between ST and language/social scores; motor association was weaker.
Sugiyama et al. (2023) [50]	Japan	Prospective birth cohort with causal mediation analysis	885	2–4 years	Parent interview; ST > 1 h/day coded as higher exposure; mean ST 2.6 ± 2.0 h/day.	Japanese Vineland Adaptive Behavior Scale, second edition at age 4: communication, daily living skills and socialization.	Higher ST at age 2 was associated with lower communication and daily living scores at age 4. Outdoor play did not mediate communication but mediated 18% of the daily-living association; outdoor play related positively to socialization.
Varadarajan et al. (2021) [51]	India	Population-based cross-sectional	718	6–60 months	Parent/guardian 7-day observation diary; mean total 2.39 h/day; excessive defined as any use at 6–23 months and >1 h/day at 24–60 months; 73% excessive.	Communication DEALL Developmental Checklist, eight developmental domains; delay defined as ≥2 skills not acquired in the age hierarchy.	Excessive ST was associated with developmental delay, especially language acquisition, expressive/receptive language and communication. Associations were present in both <2-year and ≥2-year groups.
Mulé et al. (2022) [52]	Germany	Cross-sectional pilot	49	3–5 years	Modified CHILT-III parent questionnaire	SETK 3–5 language subtests: Understanding Sentences, Formation of Morphological Rules, Phonological Working Memory for Nonwords; KiMo motor test.	Media consumption had a negative predictive effect on sentence understanding; physical inactivity was negatively related to language subtests. Some motor-language associations were positive and mixed.
Madigan et al. (2019) [53]	Canada	Three-wave longitudinal cohort	2441	2–5 years	Maternal report of weighted weekly hours	Maternal Ages and Stages Questionnaire, third edition (ASQ-III), five domains and summed developmental score.	Higher ST at 24 months predicted poorer ASQ performance at 36 months; higher ST at 36 months predicted poorer ASQ performance at 60 months. The reverse pathway was not observed.
McArthur et al. (2022) [54]	Canada	Cross-sectional cohort analysis	1994	1–3 years	Single NutriSTEP-type maternal report: ≤1 h/day *n* = 935, 2 h *n* = 725, ≥3 h *n* = 333	ASQ-III developmental milestones; MacArthur-Bates CDI language acquisition; adapted CBCL/NLSCY internalizing and externalizing behavior.	Compared with ≤1 h/day, 2 h and ≥3 h/day were associated with greater odds of behavior problems and delayed milestones; ≥3 h/day was also associated with poorer vocabulary acquisition.
**Domain B: Cognitive Development and Executive Function (*n* = 6)**							
Zhang et al. (2022) [55]	Canada	Cross-sectional analysis of quasi-experimental cohort baseline	97	36–60 months	Parent questionnaire; weighted weekday/weekend mean	Early Years Toolbox expressive vocabulary and Mr Ant working-memory task; working memory dichotomized at the median.	Total ST was not associated with expressive vocabulary but was associated with lower odds of better working memory. TV- and video-game-specific associations were not significant.
Rai et al. (2023) [56]	Canada	Cross-sectional pilot/feasibility study	44	3 years	Two-week online daily diary	Virtual working-memory span, Head-Toes-Knees-Shoulders inhibitory control, Early Years Toolkit expressive vocabulary, modified snack-delay self-control; PARCHISY parent-child interaction coding.	Total ST and show/movie/video viewing were negatively associated with working memory. Educational screen use was positively associated with vocabulary after adjustment; co-use was negatively associated with self-control in adjusted analyses. The study primarily established feasibility and preliminary effect sizes.
Schwarzer et al. (2022) [57]	Germany	Cross-sectional cohort analysis	269	2–5 years	Standardized parent questionnaires	ET 6-6-R standardized development test: body motor, hand motor, cognition, language and social-emotional percentile ranks; parent-child interaction score.	Child ST >1 h/day was associated with lower cognition, language and social-emotional percentiles; TV-specific exposure was particularly associated with cognition and language. More parent-child interaction was associated with better outcomes.
McHarg et al. (2020) [38]	United Kingdom, United States and Netherlands	Propensity score cross-sectional	416	0–2 years	Parent report of hours on a typical weekday/weekend; regular SE defined as any reported exposure.	Performance-based Prohibition/latency-to-touch inhibition; Three Boxes/multi-location search working memory; Ball Run set-shifting/cognitive flexibility.	Regular SE at 4 months predicted poorer inhibition at 14 months; no association was detected for working memory or cognitive flexibility. Continuous exposure was not linearly related to inhibition.
Huang et al. (2024) [58]	Singapore	Prospective longitudinal cohort with mediation/moderation analysis	950	1–4 years	Parent-reported weekly ST; primary average SE under age 2 and later control exposure at 3–4 years.	MRI diffusion/network topology at age 6; BRIEF-2 Emotion Regulation Index and SEARS at age 7; parent reading at age 3.	Higher infancy SE related to emotion-processing/cognitive-control network integration. The network measure mediated associations with later emotion regulation and social-emotional competence; reading buffered the network association.
Zhao et al. (2022) [39]	China	Prospective birth cohort with group-based screen-time trajectories	152	6 months–5 years	Maternal reports at eight time points. Trajectories	WISC-IV at age 72 months: FSIQ, General Ability, verbal comprehension, perceptual reasoning, Cognitive Proficiency, working memory and processing speed; Strengths and Difficulties Questionnaire (SDQ).	Compared with continued-low exposure, late- and early-increasing trajectories were associated with lower FSIQ and working memory; early-increasing exposure was associated with higher total difficulties and hyperactivity/inattention.
**Domain C: Socio-emotional development and behavior (*n* = 4)**							
Hinkley et al. (2018) [59]	Australia	Cross-sectional	575	2–5 years	Mother-reported mean total ST 2.1 h/day (TV 1.6; computer/games 0.5); outdoor play used as a physical-activity proxy.	Adaptive Social Behavior Inventory (ASBI): Express, Comply and Disrupt subscales.	TV viewing was inversely associated with compliant skills; outdoor play was positively associated with expressive and compliant skills. Computer/game time was not clearly associated.
Nurrohmah et al. (2025) [60]	Indonesia	Cross-sectional correlational	60	2–5 years	Modified validated SMALLQ	Developmental Pre-Screening Questionnaire (KPSP), personal-social domain.	Excessive ST was associated with non-age-appropriate personal-social development; 27/31 children with excessive exposure were classified as not age-appropriate.
Oflu et al. (2021) [61]	Turkey	Cross-sectional descriptive	240	2–5 years	Structured survey; intentionally sampled low (≤1 h/day) and high (≥4 h/day) groups; 98 children (40.8%) were in the high group.	Turkish-adapted parent Emotion Regulation Checklist: lability/negativity and emotion-regulation subscales.	Children with ≥4 h/day had a higher proportion in the highest lability/negativity quartile; lack of co-viewing showed a similar pattern.
Almuaigel et al. (2021) [35]	Saudi Arabia	Cross-sectional	288	1.5–5 years	Parent questionnaire	Arabic Children’s Behavior Questionnaire (CBQ): surgency, negative affect, effortful control; Sleep Disturbance Scale for Children (SDSC).	Technology use was associated with less favorable behavior scores and sleep disturbance. Owning a video-game device and TV/video-game ownership related to behavior indicators; 3–5 h/day related to worse sleep.

ASBI = Adaptive Social Behavior Inventory; ASQ-III = Ages and Stages Questionnaire, Third Edition; BRIEF-2 = Behavior Rating Inventory of Executive Function, Second Edition; CBCL = Child Behavior Checklist; CBQ = Children’s Behavior Questionnaire; CDI = Communicative Development Inventory; CELF-P2 = Clinical Evaluation of Language Fundamentals–Preschool, Second Edition; CHILT-III = Children’s Health Interventional Trial III; CPRS = Child–Parent Relationship Scale; DDST-II = Denver Developmental Screening Test II; DEALL = Developmental Eclectic Approach to Language Learning; DIBELS = Dynamic Indicators of Basic Early Literacy Skills; ET 6-6-R = Developmental Test for Children Aged 6 Months to 6 Years, Revised; FSIQ = Full Scale Intelligence Quotient; h = hours; JACDI-WG = JISH Arabic Communicative Development Inventory, Words and Gestures; JACDI-WS = JISH Arabic Communicative Development Inventory, Words and Sentences; KiMo = Kindergarten Mobile Test; KPSP = *Kuesioner Praskrining Perkembangan* (Developmental Pre-Screening Questionnaire); LENA = Language Environment Analysis; MRI = magnetic resonance imaging; *n* = sample size; NLSCY = National Longitudinal Survey of Children and Youth; PARCHISY = Parent–Child Interaction System; PPVT = Peabody Picture Vocabulary Test; SDQ = Strengths and Difficulties Questionnaire; SE = screen exposure; SEARS = Social Emotional Assets and Resilience Scales; SES = socioeconomic status; SETK 3–5 = Language Development Test for Children Aged 3–5 Years; SMALLQ = Surveillance of Digital Media Habits in Early Childhood Questionnaire; ST = screen time; TV = television; WISC-IV = Wechsler Intelligence Scale for Children, Fourth Edition.

**Table 2:** Screen Exposure Dimensions Examined Across Included Studies (*n* = 27).

Screen Exposure Dimension	Studies (*n*)	Prevalence (%)	Key Measurement Approaches
Duration/quantity	27	100%	Daily/weekly hours; guideline adherence (e.g., ≤1 h/day or ≤2 h/day)
Content quality/type	19	70%	Educational vs. entertainment; target audience; genre; age-appropriateness
Context of use	18	67%	Co-viewing vs. solitary; joint media engagement; background TV
Caregiver mediation	15	56%	Active mediation (discussion, questioning); restrictive mediation (rules, limits)
Device type	14	52%	Television; tablets; smartphones; computers; gaming devices
Background exposure	12	44%	TV on during meals, play, or other activities
Age of onset	11	41%	Age at first regular exposure; early exposure (<2 years)
Trajectories	8	30%	Stable low; stable high; early-increasing; late-increasing patterns

Percentages based on 27 total included studies. Studies commonly examined multiple dimensions simultaneously.

### 3.4. Language and Communication Development

Language and communication development was the most studied domain, examined in 17 of 27 studies (63%). The majority of studies reported associations between higher ST and delayed expressive or receptive language outcomes; however, effect magnitudes varied across study designs, samples, and measurement tools, and a minority of studies reported null or positive associations depending on content and context [36,40,48,51]. Longitudinal evidence further indicated that ST at age two and three years predicted lower communication scores at subsequent ages [53]. Moreover, in a Japanese cohort a dose-response pattern weas documented, children exposed to four or more hours of ST daily had a language delay at age 2.5 years [48]. In the Saudi context, a study found that lower SE and more reading predicted more expressive vocabulary and longer mean lengths of utterance in toddlers aged 17–36 months, while in younger toddlers (12–16 months), interactive joint media engagement and verbal co-engagement predicted larger receptive vocabulary [36]. Reverse causality is a particularly important consideration in cross-sectional studies; developmental difficulties may precede increased SE rather than result from it.

However, content quality and context substantially moderated these associations. High-quality educational programming, particularly when accompanied by active caregiver mediation, was associated with vocabulary learning [15]. Conversely, adult-directed entertainment content and background television associated with poorer language outcomes [18,46]. A study in Pakistan documented in 318 children aged 2.5–5 years that daily screen use was significantly associated with delayed language development [46], while another study documented, using ecological momentary assessment in 220 families, that higher ST was associated with reduced the quantity and quality of parent-child verbal interaction in children aged 12–36 months, representing a plausible displacement of language learning opportunities [18]. Joint media engagement emerged as a key protective factor, adult-child co-use during digital media was associated with improved children’s learning outcomes, with the largest effects observed for language. Active co-viewing, involving discussion, questioning, and connecting content to children’s experiences, proved substantially more beneficial than passive co-presence [36,43].

### 3.5. Cognitive Development and Executive Function

Cognitive development and executive function outcomes were examined in 6 of 27 studies (22%). Across those studies, associations between higher ST and poorer performance on attention, inhibitory control, working memory, and general cognitive ability were predominantly negative; however, the limited number of studies and the heterogeneity of cognitive assessment tools preclude strong conclusions about the size or consistency of these associations [39,56,57]. Longitudinal trajectory studies provided notable evidence. Zhao et al. (2022) found in a prospective Chinese birth cohort that early-increasing and late-increasing ST trajectories from 6 to 72 months were associated with lower full-scale IQ and working memory scores at age six compared to a stable low-exposure group, with early-increasing trajectories also predicting higher total difficulties and hyperactivity/inattention [39]. Another study demonstrated in a UK longitudinal cohort that infant SE at 6–18 months predicted poorer inhibitory control at age three, with effects specific to inhibition rather than broader executive function constructs, suggesting impacts on specific neurodevelopmental processes [38].

Content quality and context moderated cognitive associations. A study in Germany found that ST at 24 months predicted lower cognitive scores at 4 years, lower language scores, and lower social-emotional development scores, even after controlling for sociodemographic factors [57]. Another study reported in a Chinese sample that high ST was associated with reduced odds of age-appropriate working memory [39], while Rai et al. (2023) found that overall SE predicted lower working memory scores, while educational screen use was associated with better vocabulary, highlighting the moderating role of content quality [56]. A Singaporean cohort showed that associations between ST and cognitive outcomes were partially explained by brain network connectivity, providing a possible neurobiological account of the observed associations [58].

### 3.6. Socio-Emotional and Behavioral Development

Socio-emotional and behavioral outcomes were examined in 4 of 27 studies (15%). Three of those studies reported associations between higher ST and increased behavioral problems, emotional lability, or reduced prosocial behavior; the fourth study reported null associations for certain outcomes. Given the small number of studies, these findings should be interpreted with caution [35,59,60,61].

In the Saudi context, preschool children with high technology use demonstrated elevated behavioral and socio-emotional problem scores compared to those with lower use [35]. Video-game device ownership was independently associated with poorer effortful control, and television or video-game ownership with poorer negative affect, even after controlling for sociodemographic variables. Unmediated screen use showed the strongest associations with behavioral difficulties.

In a Turkish sample, children in the highest ST quartile had higher emotional lability scores than those in the lowest quartile [61]. An Australian longitudinal study reported that television viewing at age two years predicted lower social skills at age four years, with effects persisting after adjustment for baseline social competence and sociodemographic factors [59]. An Indonesian study similarly found that excessive screen use was associated with delays in age-appropriate personal-social development [60], raising concerns that prolonged SE may displace opportunities for social interaction and skill acquisition.

Caregiver mediation was associated with more favorable outcomes across the socio-emotional domain. Almuaigel et al. (2021) as described above [35], further found that unmediated exposure, without caregiver co-viewing, discussion, or content guidance was associated with elevated behavioral problem scores, while Oflu et al. (2021) similarly demonstrated that unmediated exposure; without caregiver co-viewing, discussion, or content guidance was associated with elevated emotional lability scores, while mediated screen use showed attenuated negative associations [61]. Table 3 summarizes key findings regarding associations between SE dimensions and developmental outcomes.

### 3.7. Cross Cutting Themes and Possible Pathways

Several themes emerged across developmental domains. First, duration alone proved insufficient for understanding SE effects; content quality, context, and caregiver mediation consistently shaped the strength and direction of associations [36,43,61]. Second, early exposure and increasing trajectories appeared particularly consequential, suggesting sensitive periods and cumulative effects [38,39,48]. Third, displacement of enriching activities; parent-child conversation, physical activity, creative play, and sleep has been proposed as a plausible mechanism consistent with the observational associations reported, however, this displacement hypothesis has not been experimentally established in the included studies [18,59]. Fourth, the Saudi Arabian and regional studies [35,36] revealed patterns broadly consistent with international findings while highlighting context-specific factors: high smartphone and tablet use, a gap in Arabic-language educational content, and the need for culturally adapted assessment and intervention approaches. Additional regional evidence from countries including Pakistan [46], India [41,51], Indonesia [60], Turkey [61], and Denmark [40] confirmed that the multidimensional nature of SE effects transcends cultural and socioeconomic contexts.

## 4. Discussion

This scoping review synthesized evidence from 27 studies demonstrating that SE in early childhood is a multidimensional phenomenon whose developmental associations vary depending on content quality, context of use, caregiver mediation, device characteristics, timing, and trajectories and not duration alone. While duration was the only dimension assessed in all 27 included studies, content quality was examined in 19 studies (70%), context in 18 (67%), and caregiver mediation in 15 (56%). This pattern suggests the field is beginning to move towards multidimensional assessment, but duration-based measurement remains dominant and operationalization of non-duration dimensions remains heterogeneous across studies [6,12].

### 4.1. The Multidimensional Framework in Practice

The evidence demonstrates that while excessive ST (typically > 2 h/day for young children) shows robust negative associations with language, cognitive, and socio-emotional development [19,22,39], these associations are substantially moderated by other dimensions. High-quality educational content co-viewed with actively engaged caregivers can support learning [7,19]. Conversely, even modest amounts of background television or exposure to age-inappropriate content predict negative outcomes independently of total duration [17,18].

This pattern aligns with frameworks emphasizing the “three Cs” of media effects: content, context, and child characteristics [8,9]. Content quality, including age-appropriateness, educational design, pacing, and linguistic characteristics may influence whether SE provides learning opportunities or cognitive overload. Context of use, whether screens are used solitarily or socially, during enriching routines or before sleep, with or without caregiver mediation, may shape whether ST displaces or complements developmental experiences [17,62].

Longitudinal trajectory studies [38,39] contribute a temporal dimension to this evidence base. Children following early-increasing trajectories were associated with poorer outcomes than children with stable low or stable moderate trajectories, based on eight longitudinal studies; while the consistency of this pattern across studies is notable, the limited number of trajectory-specific studies precludes strong conclusions about developmental risk. These findings suggest that the timing and pattern of SE introduction may be relevant alongside absolute duration, though further longitudinal evidence is needed to clarify the mechanisms involved.

The displacement of enriching activities such as parent-child conversation, physical activity, creative play, and sleep has been proposed as a plausible mechanism consistent with the observational associations reported; however, this displacement hypothesis has not been experimentally established in the included studies [17,18]. Studies in which screens appeared to displace such activities were associated with poorer developmental outcomes, while studies in which caregivers actively mediated exposure and supplemented rather than supplanted enriching activities reported more benign or beneficial associations [19,20]. It should be noted, however, that the observational nature of most included studies means directionality cannot be assumed, children with developmental difficulties may also receive greater SE as a management strategy, and this reverse causality pathway should be considered when interpreting the evidence. Furthermore, as no formal risk-of-bias assessment was conducted as part of this scoping review, the methodological quality of the studies informing this proposed mechanism was not systematically evaluated, and causal conclusions cannot be drawn.

### 4.2. The Saudi Arabian and Middle Eastern Context

The two Saudi Arabian studies included in this review [35,36] revealed patterns broadly consistent with international research while highlighting important region-specific considerations. Almuaigel et al. (2021) found that unmediated technology use was associated with socio-emotional and behavioral problems in Saudi preschoolers [35], aligning with meta-analytic evidence from Western contexts [19,63]. Similarly, a study documented that Saudi toddlers frequently exceeded WHO ST recommendations, with high mobile device use and limited exposure to Arabic-language educational content associated with language development delays [36].

The evidence synthesized in this review derives predominantly from global studies; only two of 27 included studies originated from Saudi Arabia, and broader MENA representation was similarly limited. The following observations about the regional context should therefore be understood as contextual hypotheses and research priorities motivated by this knowledge gap, not as conclusions derived from regional evidence. The hot, arid climate characteristic of the Arabian Peninsula limits outdoor play opportunities for substantial periods of the year, increasing the probability of indoor screen-based activities during early childhood. Family media dynamics are further shaped by high device penetration rates, multi-generational household structures, and predominantly Arabic-language media consumption patterns that may influence content quality and co-viewing behaviors differently from Western contexts [64]. The linguistic landscape is particularly complex: Modern Standard Arabic differs substantially from spoken colloquial dialects, and many families use multiple languages, complicating content selection and language development pathways [7,36]. The documented gap in high-quality, developmentally appropriate, Arabic-language content for young children represents a structural barrier to healthy screen use [15].

Saudi Arabia’s Vision 2030 initiative, with its emphasis on early childhood development and education reform, provides important policy momentum. However, the translation of international evidence into locally relevant guidance requires culturally adapted tools and norms. Existing assessment instruments, predominantly developed in Western contexts, may not adequately capture regional patterns of device use, content preferences, extended family caregiving arrangements, or cultural values around technology and child-rearing [37,65]. These gaps directly motivate the development of a validated, multidimensional SE assessment tool for Saudi Arabia and the Middle East region.

### 4.3. Implications for Practice and Policy

The evidence base reviewed supports several practical implications. For families, guidance should move beyond simple duration limits to emphasize content quality, co-viewing practices, and active mediation [20,27]. Parents need support in selecting age-appropriate, educational content; strategies for interactive co-viewing; and approaches for setting appropriate limits while maintaining positive parent-child relationships.

For practitioners; pediatricians, early childhood educators, and speech-language therapists screening tools should capture not only duration but also content, context, and caregiver mediation patterns [37,65]. Multidimensional screening is particularly important in high-risk contexts: children with early language delay, behavioral difficulties, or developmental concerns may be especially vulnerable to high or unmediated SE [23,35].

At the policy level, efforts should address structural factors: supporting development of high-quality, culturally appropriate Arabic-language educational content for young children; integrating media literacy and healthy screen use into early childhood education curricula; and ensuring equitable access to alternative enrichment activities [20,65]. In Saudi Arabia and the Middle East, Vision 2030’s early childhood development priorities could incorporate evidence-based SE guidance, including the development and validation of culturally adapted assessment tools, training of healthcare providers in multidimensional assessment, and public awareness campaigns emphasizing quality, co-viewing, and active mediation over simple duration limits.

### 4.4. Limitations of This Scoping Review

Several limitations warrant consideration. First, the included studies employed diverse methodologies, measurement approaches, and definitions of SE, limiting direct comparability. Screen time was measured via parental report in most studies, introducing potential recall bias and social desirability effects [6,37]. Few studies employed objective passive-sensing measurement, and fewer still captured real-time content and context. The methodological limitations of the primary literature also warrant explicit acknowledgement: reliance on parent-report questionnaires, the absence of objective exposure measures, cross-sectional designs in the majority of studies, and variable adjustment for potential confounders (including parental education, socioeconomic status, parental mental health, family routines, and parent–child interaction quality) collectively limit the strength of causal inference from the synthesized evidence. The review did not formally distinguish adjusted from unadjusted associations, which is a further interpretive limitation. Second, the majority of studies were cross-sectional, precluding causal inference. While longitudinal designs provide stronger temporal evidence [38,39], reverse causality remains plausible: children with developmental delays may be given more ST by parents seeking to manage challenging behavior [66]. This bidirectional possibility should be considered when interpreting all domain-level associations reported in this review.

Third, the evidence base is heavily weighted toward Western, educated, industrialized, rich, and democratic (WEIRD) populations. Only two of 27 included studies were conducted in Saudi Arabia, and broader Middle Eastern representation was limited [64,67]. Fourth, Restriction to peer-reviewed literature may have excluded relevant evidence from grey literature sources, including government health reports, policy documents, and practice guidelines, which may be particularly consequential for the Saudi Arabian and MENA context where peer-reviewed primary data are limited. Although 26% of the records could not be retrieved, this may reflect limited institutional access to paywalled journals and inability to obtain full texts directly from corresponding authors, representing a potential limitation on the comprehensiveness of the synthesized evidence. Fifth, the field lacks consensus on measurement approaches for SE dimensions beyond duration; content quality, caregiver mediation, and context of use remain challenging to quantify, with existing measures showing variable reliability and validity [6,37]. Sixth, the evidence base was heavily weighted towards language and communication outcomes (63% of included studies), with cognitive and socio-emotional domains represented by markedly fewer studies (22% and 15%, respectively); conclusions drawn from these latter domains should therefore be interpreted with caution and regarded as preliminary pending a more balanced evidence base. Finally, as a scoping review rather than a systematic review or meta-analysis, this review maps the landscape of evidence without quantitative synthesis of effect sizes.

### 4.5. Future Research Directions

Future research should prioritize longitudinal designs with ecologically valid, multidimensional measurement of SE capturing content, context, and caregiver mediation in real time [6,12]. Experimental and quasi-experimental studies examining specific dimensions (content quality, caregiver mediation strategies, device types) can strengthen causal inference while remaining ethically feasible [19]. Research in diverse cultural, linguistic, and socioeconomic contexts is essential for identifying universal versus context-specific patterns [64,67].

In the Saudi Arabian and Middle Eastern context specifically, population-based longitudinal studies, development and validation of culturally adapted assessment tools, and trials of culturally appropriate parent-mediated interventions are urgently needed [36,65]. Finally, mechanistic research employing neuroimaging and psychophysiological methods can elucidate the pathways through which SE may be associated with developmental process in the brain, informing more targeted interventions [8,9].

## 5. Conclusions

This scoping review demonstrates that SE in early childhood is a multidimensional phenomenon whose developmental associations extend beyond duration alone, though duration remains the only dimension assessed across all 27 included studies. The evidence base is beginning to move towards multidimensional assessment, with content quality, context, and caregiver mediation increasingly examined, but measurement of these dimensions remains inconsistent. While excessive ST shows the most robust negative associations with language development (examined in 17 of 27 studies), associations with cognitive development (6 studies) and socio-emotional outcomes (4 studies) are based on fewer studies and should be interpreted with greater caution. Across domains, these associations appear to be modified by what children watch, with whom, and how caregivers engage. Co-viewing with actively engaged caregivers has been associated with positive learning outcomes; background television, inappropriate content, and unmediated exposure are associated with poorer outcomes across the developmental domains examined.

The Saudi Arabian and Middle Eastern context presents several contextual factors, including high device penetration, a gap in Arabic-language educational content, and distinctive cultural patterns of family media use that represent important research priorities. Given that only two of the included studies were conducted in Saudi Arabia and broader MENA representation was limited, these factors should be understood as contextual hypotheses warranting direct empirical investigation rather than conclusions directly established by this review. The global evidence synthesized here provides the conceptual scaffold for developing a culturally adapted, multidimensional SE assessment tool for Saudi and MENA contexts, with the identified dimensions (duration, content quality, context of use, caregiver mediation, device type, and trajectory) defining the construct domains any such instrument must capture. Cultural adaptation would further require Arabic translation and validation, incorporation of regionally relevant content categories, and normative data from primary Saudi and MENA studies. This review is explicitly positioned as the evidence-mapping step that precedes and informs that instrument development process, not as sufficient evidence to complete it alone.

## Figures and Tables

**Figure 1:**
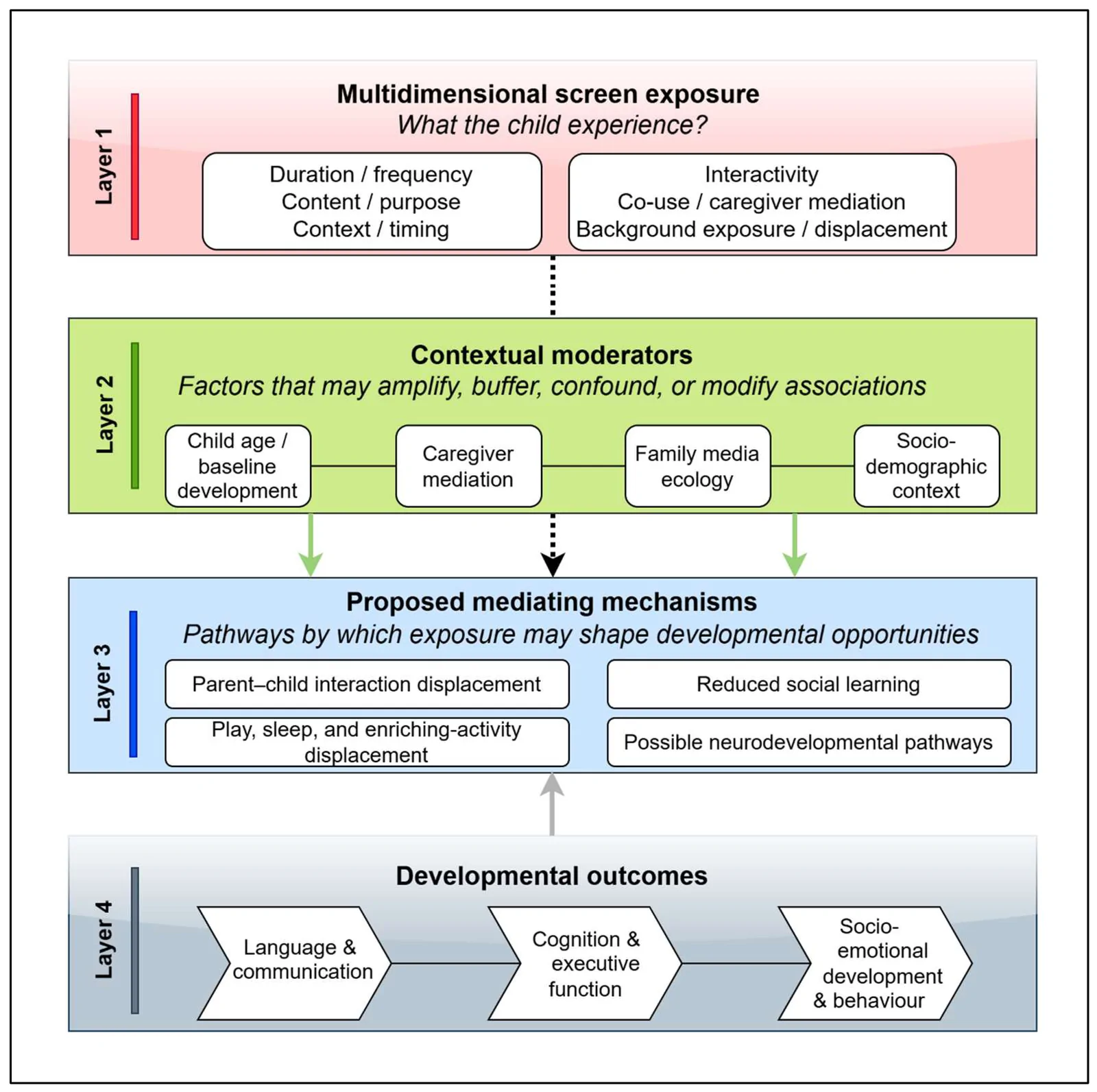
A proposed multidimensional framework for understanding screen exposure and early childhood development. Layer 1 defines the dimensions of screen exposure; Layer 2 identifies contextual modifying factors that amplify or buffer associations; Layer 3 proposes putative mechanisms linking exposure to developmental outcomes, pending formal mediation testing in future research; Layer 4 maps the three developmental outcome domains synthesized in this review.

**Figure 2:**
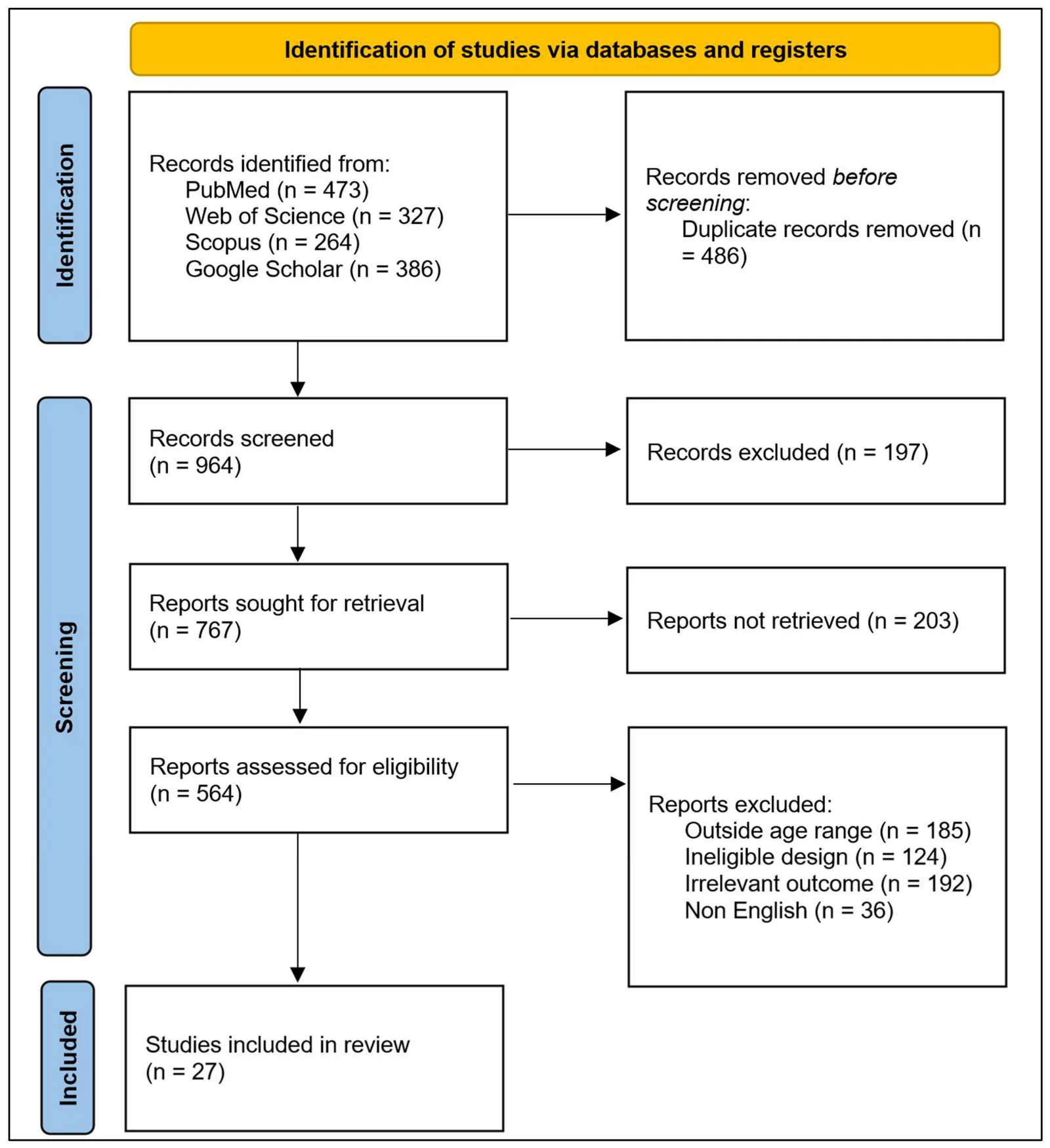
PRISMA-ScR flow diagram using the detailed screening counts reported in the supplied manuscript.

**Table 3:** Summary of Developmental Outcomes by Domain and Screen Exposure Dimension.

Domain	Screen Dimension	Direction	Key Finding
Language & Communication	Duration (excessive)	(−)	Consistently associated with vocabulary deficits, expressive/receptive language delays
	Content quality	(+/−)	Educational, child-directed content supports language when co-viewed; adult-directed entertainment shows negative associations
	Co-viewing/joint engagement	(+)	Active co-viewing with verbal interaction is protective; passive co-presence shows minimal benefit
	Background TV	(−)	Reduces parent-child verbal interaction and language input quality
Cognitive & Executive Function	Duration (excessive)	(−)	Associated with attention problems, reduced inhibitory control, lower working memory
	Early-increasing trajectories	(−)	Lower IQ scores and executive function deficits at school age
	Content type	(Mixed)	Educational content may support specific skills; fast-paced/violent content impairs attention and self-regulation
	Context (meals, bedtime)	(−)	Associated with cognitive deficits, likely via disrupted family interaction
Socio-Emotional/Behavioral	Duration (excessive)	(−)	Linked to emotional lability, behavioral problems, and reduced prosocial behavior
	Unmediated exposure	(−)	Strongest predictor of behavioral and emotional problems across studies
	Content type (violent/inappropriate)	(−)	Associated with aggression and conduct problems
	Active caregiver mediation	(+)	Buffers negative socio-emotional effects; promotes emotional regulation

(+) indicates a positive/beneficial association; (−) indicates a negative/harmful association. Associations reflect predominant patterns across multiple studies; individual study findings may vary.

## Data Availability

Data sharing is not applicable to this article as no new datasets were generated or analyzed during the current study. All analyzed data are included in this published article. The protocol for this review was registered retrospectively on the Open Science Framework (OSF) on (https://doi.org/10.17605/OSF.IO/UQKVD).

## Supplementary Materials

The following supporting information can be downloaded at: https://www.mdpi.com/article/10.3390/healthcare14193206/s1, Supplementary File S1: Full Electronic Search Strategy.
