# Supplementary material for: Beyond Screen Time: A Scoping Review of Screen Exposure and Early Childhood Development
Source: Healthcare (Basel). 2026 Sep 28;14(19):3206. doi: 10.3390/healthcare14193206 (PMC13664933; doi:10.3390/healthcare14193206)
Supplement: Supplementary file 1 [file healthcare-14-03206-s001.zip › healthcare-4554891-supplementary.pdf]

Supplementary File S1: Full Electronic Search Strategy

Search Concept Blocks

Search terms were organized into four conceptual blocks combined with Boolean AND operators. All terms within each block were combined with OR.

Block 1 — Screen Exposure (SE)

```
"screen time" OR "screen exposure" OR "digital media" OR "mobile
device" OR
"tablet use" OR "smartphone use" OR "television" OR "TV viewing" OR
"electronic media" OR "media use" OR "social media" OR "video games" OR
"streaming" OR "online video" OR "YouTube" OR "passive screen" OR
"interactive media"
```

Block 2 — Population (Early Childhood, 0–5 years)

```
"early childhood" OR "preschool" OR "preschool children" OR "toddler"
OR
"toddlers" OR "infant" OR "infants" OR "young children" OR "0-5 years"
OR
"under five" OR "kindergarten" OR "early years"
```

Block 3 — Developmental Outcomes

```
"language development" OR "language delay" OR "speech development" OR
"vocabulary" OR "cognitive development" OR "executive function" OR
"attention" OR "working memory" OR "socio-emotional development" OR
"social-emotional" OR "behavioral outcomes" OR "behavior problems" OR
"developmental outcomes" OR "child development" OR "communication
skills" OR "emotional regulation" OR "school readiness" OR "physical
development" OR "developmental delay" OR "physical ability"
```

Block 4 — Regional Block (MENA; applied selectively)

```
"Saudi Arabia" OR "Middle East" OR "MENA" OR "Arab" OR "Gulf" OR
"United Arab Emirates" OR "UAE" OR "Jordan" OR "Egypt" OR "Lebanon" OR
"Kuwait" OR "Qatar" OR "Bahrain" OR "Oman" OR "Middle Eastern"
```

Full Search Strings by Database

PubMed (MEDLINE)

```
(("screen time"[tiab] OR "screen exposure"[tiab] OR "digital
media"[tiab]OR "mobile device"[tiab] OR "tablet use"[tiab] OR
"smartphone use"[tiab] OR "television"[tiab] OR "TV viewing"[tiab] OR
"electronic media"[tiab] OR "media use"[tiab] OR "social media"[tiab]
OR "video games"[tiab] OR "streaming"[tiab] OR "interactive
media"[tiab])
AND
("early childhood"[tiab] OR "preschool"[tiab] OR "preschool
children"[tiab] OR "toddler"[tiab] OR "infant"[tiab] OR "young
```

```
children"[tiab] OR "child, preschool"[MeSH] OR "child
development"[MeSH])
AND
("language development"[tiab] OR "language delay"[tiab]
OR "cognitive development"[tiab] OR "executive function"[tiab]
OR "socio-emotional"[tiab] OR "social-emotional"[tiab]
OR "behavioral outcomes"[tiab] OR "developmental outcomes"[tiab]
OR "child development"[tiab] OR "communication skills"[tiab] OR
"physical development" [tiab] OR "emotional regulation"[tiab] OR
"school readiness"[tiab]))
AND ("2016/01/01"[pdat] : "2026/07/31"[pdat])
AND (English[lang])
```

Scopus

```
TITLE-ABS-KEY(
  ("screen time" OR "screen exposure" OR "digital media" OR "mobile
device"
   OR "tablet use" OR "smartphone use" OR "television" OR "TV viewing"
   OR "electronic media" OR "media use" OR "social media" OR "video
games" OR "streaming" OR "interactive media")
  AND
  ("early childhood" OR "preschool" OR "toddler" OR "infant"
   OR "young children" OR "kindergarten")
  AND
  ("language development" OR "language delay" OR "cognitive
development" OR "physical development" OR "executive function" OR
"socio-emotional" OR "social-emotional" OR "behavioral outcomes" OR
"developmental outcomes" OR "communication skills" OR "emotional
regulation" OR "school readiness")
)
AND PUBYEAR > 2015
AND PUBYEAR < 2027
AND ( LIMIT-TO ( LANGUAGE , "English" ) )
```

Web of Science (WoS) — Core Collection

```
TS=("screen time" OR "screen exposure" OR "digital media" OR "mobile
device"
    OR "tablet use" OR "smartphone use" OR "television" OR "TV viewing"
    OR "electronic media" OR "media use" OR "social media" OR "video
games"
    OR "streaming" OR "interactive media")
AND TS=("early childhood" OR "preschool" OR "toddler" OR "infant"
        OR "young children" OR "kindergarten")
AND TS=("language development" OR "language delay" OR "cognitive
development" OR "physical development" OR "executive function" OR
"socio-emotional" OR "social-emotional" OR "behavioral outcomes" OR
"developmental outcomes" OR "communication skills" OR "emotional
```

```
regulation" OR "school readiness")
AND PY=(2016-2026)
AND LA=(English)
```

Google Scholar (Supplementary Source)

Google Scholar does not support complex Boolean syntax. The following keyword string was entered in the search box. Results were screened manually for the first 200 records sorted by relevance, as per the Peer Review of Electronic Search Strategies (PRESS) guidance for supplementary grey-literature sources.

```
"screen time" OR "screen exposure" OR "digital media"
"early childhood" OR "preschool children" OR "toddler"
"language development" OR "cognitive development" OR "developmental
outcomes" OR "physical development"
After 2015
```
